# Correction: Protocol: The role of defunctioning stoma prior to neoadjuvant therapy for locally advanced colonic and rectal cancer-A systematic review

**DOI:** 10.1371/journal.pone.0308240

**Published:** 2024-07-30

**Authors:** Mina Mesri, Louise Hitchman, Marina Yiasemidou, Aaron Quyn, David Jayne, Ian Chetter

The third author’s name is spelled incorrectly. The correct name is: Marina Yiasemidou. The correct citation is: Mesri M, Hitchman L, Yiasemidou M, Quyn A, Jayne D, Chetter I (2022) Protocol: The role of defunctioning stoma prior to neoadjuvant therapy for locally advanced colonic and rectal cancer-A systematic review. PLOS ONE 17(9): e0275025. https://doi.org/10.1371/journal.pone.0275025.
