# Supplementary material for: Protocol: The role of defunctioning stoma prior to neoadjuvant therapy for locally advanced colonic and rectal cancer-A systematic review
Source: PLoS One. 2022 Sep 22;17(9):e0275025. doi: 10.1371/journal.pone.0275025 (PMC9498940; doi:10.1371/journal.pone.0275025)
Supplement: S1 Appendix — (DOCX) [file pone.0275025.s002.docx]

**S1 Appendix.**

***Table 1*: Search terms**: the exact terms stated below were used across all search databases

| Search set |  | Search terms (Title) |
| --- | --- | --- |
| 1 |  | "colonic cancer" OR "colon cancer" OR "rectal cancer" OR "colorectal cancer" OR "rectal malignancy" OR "colonic malignancy" OR "colorectal malignancy" |
| 2 |  | "neoadjuvant" OR "pre-operative chemotherapy" OR "pre-operative radiotherapy" OR "pre-operative chemoradiotherapy" OR "preoperative radiotherapy" OR "preoperative chemotherapy" OR "preoperative chemoradiotherapy" |
| 3 |  | 1 and 2 |
| Limits |  | Adults, English language, 2002-2020 time of publication |
